# Changes of Airway Space and Flow in Patients Treated with Rapid Palatal Expander (RPE): An Observational Pilot Study with Comparison with Non-Treated Patients

**DOI:** 10.3390/jcm14124357

**Published:** 2025-06-18

**Authors:** Paolo Faccioni, Alessia Pardo, Giorgia Matteazzi, Erika Zoccatelli, Silvia Bazzanella, Elena Montini, Fabio Lonardi, Benedetta Olivato, Massimo Albanese, Pietro Montagna, Giorgio Lombardo, Miriana Gualtieri, Annarita Signoriello, Giulio Conti, Alessandro Zangani

**Affiliations:** Section of Oral and Maxillofacial Surgery, Department of Surgical Sciences, Dentistry, Gynecology and Pediatrics, University of Verona, 37124 Verona, Italy; paolo.faccioni@univr.it (P.F.);

**Keywords:** rapid palatal maxillary expansion, airway volume, airway flow, cone beam computed tomography

## Abstract

**Background/Objectives**. With a rapid palatal expander (RPE) is reported to be effective in increasing the volume of nasal cavities, with a restoration of physiological nasal airflow. The purpose of this retrospective clinical study was to evaluate, using Cone Beam Computed Tomography (CBCT), the volumetric changes and airflow velocity changes in the nasal cavities, retro-palatal and retro-glossal airways, resulting from the use of RPE with dental anchorage (group A), also comparing these data with patients non treated with RPE (group B). **Methods**. Sixteen subjects (aged 9.34 years) with transverse maxillary deficiency and unilateral posterior crossbite were treated with RPE with dental anchorage. Additionally, 8 patients (aged 11.11 years) with juvenile idiopathic arthritis, who did not undergo any orthodontic treatment, were selected as a control group. Expansion was performed until overcorrection was achieved, and the device was left in place for 6 months as fixed retention, followed by another 6 months of night-time removable retention. From the retrospective evaluation, all patients presented two CBCT scans at baseline (T0) and 1-year follow-up (T1). The 3D-Slicer software was used for each CBCT to measure the nasal (VN), retropalatal (VRP), and retroglossal (VRG) volumes, while an iterative Excel spreadsheet allowed for a pilot approximated modeling and calculation of airway flow-related data. **Results**. Regarding mean age, a statistically significant difference (*p* = 0.01 *) was found between groups, suggesting that group B is closer to the pubertal growth peak. Analysis between T0 and T1 revealed: (i) a statistically significant increase for volumes VN, VRP and VRG in group A; (ii) a statistically significant increase for VN in group B; (iii) a statistically significant decrease for all variables related to airflow velocity in both groups. Furthermore, comparison between group A and B, regarding variations between T0 and T1, found a statistically significant difference only for VN. **Conclusions**. Within the limitations of this pilot evaluation, the treatment with RPE revealed promising outcomes for retro-palatal, retro-glossal and nasal volumes, together with clinical changes in airflow velocities.

## 1. Introduction

Transversal maxillary deficiency is the most common skeletal alteration affecting the maxilla, with a prevalence among patients under 18 years ranging from 2.7% to 23.3% [1].

In presence of transverse maxillary deficiency, patients are forced to shift the mandible to seek the maximum number of occlusal contacts and the greatest inter-cuspidation (especially in canine contact). If not corrected early, it can lead to asymmetric mandibular growth [2]: as the transverse dimension is the first to stabilize, the status of primary occlusion can influence the development of permanent occlusion, compromising both sagittal and vertical development of maxilla and teeth [3,4].

The etiology is complex and includes the following genetic, environmental, and behavioral factors [5]: heredity; inadequate dental arch length; excessive retention or early loss of primary teeth; presence of supernumerary teeth; bad oral habits, e.g., thumb sucking or pacifier use beyond 36 months of age, oral breathing and certain types of swallowing) [6,7]; anteroposterior skeletal discrepancy between dental arches; cleft lip or palate [8]; short lingual frenulum. Among these factors, oral breathing with chronic respiratory disorders, seem to influence the development of maxilla and predispose to transverse contraction with an association rate of up to 47% of cases [6].

Diagnosis is based on an initial clinical approach, confirmed by subsequent photographic and radiographic examination, and analysis of study models in Class I relationship [8]. This clinical evaluation considers [1,3] the shape and symmetry of the maxillary dental arch, the shape of the palatal vault, the occlusion, the predominant modality of respiration (oral or nasal) and the width of the “buccal corridors”. Regarding radiographic evaluation, the recent use of Cone Beam Computed Tomography (CBCT) allows a more accurate representation of craniofacial regions, with a visual assessment of the spatial relationship between jaws and the evaluation of the upper airways. CBCT nowadays represents a reliable, easily accessible, relatively economical and safe method in the dental field [9,10,11,12]: the patient is exposed to a low overall dose of radiation, and the format DICOM (Digital Imaging and Communications in Medicine) is compatible with high resolution, allowing scan of the patient in an upright position [6], which is recommended for evaluating the upper airways.

One of the main manifestations of transverse maxillary deficiency is the posterior crossbite, bilateral or unilateral (80% of cases) [5,13], with skeletal, dental, or dento-skeletal etiology. In general, three basic approaches of treatment are considered for managing posterior crossbite in primary dentition [2,3,13]:

- correction and monitoring of bad habits contributing to the etiology:
- removal of dental interferences or creation of a cusp guide, to prevent the patient from chewing on the side with the functional crossbite (usually considered for unilateral crossbites associated with functional shifts with canine guidance);
- actively expansion of the contracted maxillary arch using removable or fixed appliances.

As posterior crossbite does not often show spontaneous correction the early treatment with palatal expander [14] aims to promote a proper dento-skeletal relationship, improve masticatory function, and establish a proper condylar-fossa relationship. Based on the number of activations performed daily, expansion protocols are distinguished in rapid protocol (two expansions daily, one in the morning and one in the evening) and slow protocol (two expansions per week). Even if in both cases effective orthopedic expansion of the maxilla can be observed, most of the literature [9,15,16] reports slightly better results with the rapid palatal expander (RPE).

RPE is generally considered an effective method for the expansion of transverse diameters of the maxilla: as maxillary bones constitute about 50% of the anatomical structures of the nasal cavity, treatments altering the morphology of the maxillary dental arch can also affect the geometry and function of the nasal cavity [17]. An increase in width of the nasal cavity can be observed immediately after expansion, particularly evident for the nasal floor adjacent to the palatine suture, which follows a lateral movement, along with a straightening of the nasal septum. Furthermore, many studies have shown an increase in the volume of nasal cavities after treatment with RPE, with a restoration of physiological nasal airflow [18]. Nevertheless, several authors still debate about its influence on nasal airways [19]: there is still a significant controversy regarding the effects of RPE in increasing size and reducing resistance to airflow for the nasal cavity and the retro-palatal and retro-glossal areas [1,20].

In the light of these considerations, the aim of this observational clinical study was to evaluate, using CBCT, the volumetric changes and airflow velocity changes in the nasal cavities, retro-palatal and retro-glossal airways, resulting from the treatment with a rapid palatal expander (RPE) with dental anchorage (group A), also comparing these data with patients non treated with RPE (group B).

## 2. Materials and Methods

### 2.1. Study Design

An observational clinical retrospective study was conducted in 2025, on patients who had been referred and treated (between 2018 and 2024) at the Dental and Maxillo-Facial Surgery Clinic at the University of Verona, with at least one year of follow-up.

The study was approved by the University Institutional Review Board of Verona. (protocol Prog. 70,252 CESC, date 30 October 2018). The nature and aim of the study, together with the anonymity in the scientific use of data (also including the processing of sensitive data and pictures), were clearly presented in a written informed consent form, and signed by every patient. All procedures accorded with Helsinki Declaration and good clinical practice guidelines for research on human beings.

### 2.2. Inclusion and Exclusion Criteria

Patients of the group A were included in the study for:

○ age between 8 and 12 years;
○ diagnosis of transverse maxillary deficiency characterized by unilateral posterior cross-bite (transverse dental discrepancy between 5 mm and 6 mm), based on photographs, X-rays (CBCT) and study models [10,11,12];
○ pre-pubertal skeletal maturity stage, evaluated according to the cervical vertebral maturation (CVM) staging [21,22] as below cervical stage 3 (CS3);
○ treatment with RPE;
○ complete clinical documentation, including CBCT, photographs, study models, and anamnesis forms, at baseline (T0) and at 1-year follow-up (T1);
○ good oral hygiene.

Patients of group B were included in the study for:

○ age between 8 and 12 years;
○ complete clinical documentation, including CBCT scans of the facial structure and anamnesis forms, at T0 and at T1; in this group of patients, CBCT was performed for diagnosis and follow-up of juvenile idiopathic arthritis [23,24] affecting temporomandibular joint; these patients were regularly followed at the Pediatric Rheumatology Department at G.B. Rossi Hospital and at the Civile Maggiore Hospital in Verona.

The exclusion criteria for both groups included:

○ patients who had already undergone orthodontic treatment;
○ history of nasal or tonsils surgery;
○ genetic diseases or conditions interfering with treatment;
○ craniofacial syndromes;
○ cleft lip and palate;
○ significant skeletal anomalies or facial asymmetries;
○ severe adeno-tonsillar hypertrophy;
○ bilateral cross-bite;
○ post-pubertal developmental stage.

For group A, dental anomalies, temporomandibular joint disorders and periodontal defects (such as dehiscences and fenestrations affecting teeth used for anchorage) were also excluded. For group B, history of mid-facial trauma or surgery were also excluded.

### 2.3. Orthodontic Treatment with RPE for Group A

During the first visit, at T0, anamnesis forms, extra-oral and intra-oral photographs were taken for each patient, and alginate impressions of the dental arches were made for the fabrication of study models.

Furthermore, CBCT of the facial skeleton, panoramic radiograph, and lateral cephalometric radiography were performed. A cephalometric tracing was conducted according to the European Board of Orthodontics (E.B.O.) [22], along with the analysis of the cervical vertebral maturation stage based on the CVM staging system [10,21,22]. The Wilson curve and the width of the palatal vault were analyzed on the study models to determine the presence of a posterior cross-bite of either skeletal or dental etiology.

The transverse discrepancy was measured on the models using a pair of fine-pointed calipers (with a precision of 0.1 mm) as the difference between the mesiopalatal cusp of the right and left upper first molars and the central fossa of the right and left lower first molars. Considering the post-treatment relapse [15], the necessary correction was supplemented with overcorrection of the defect itself to bring the buccal slopes of the palatal cusps of the upper sixth molars close to the lingual slopes of the buccal cusps of the lower molars.

Patients were treated using two different types of RPE devices, the McNamara type and the Hyrax type (both made by the same dental laboratory inside the Dental Clinic of the University of Verona ), which use the same type of median screw (see Appendix A.1). Given the evidence [25,26,27,28,29,30,31,32] indicating no significant differences between the two devices, as both involve dental anchorage, it was decided to consider this group as entire (group A).

Patients were treated until the achievement of the correct transverse width of the dental arches: they were subsequently monitored after one week and at the end of the activation phase. Once the correct transverse dimensions were achieved, the expander screw was locked in place using a 0.01-inch metal ligature and flowable composite. Intra-oral photographs were then taken according to the photographic protocol used. The appliance was kept in place for 6 months as a fixed retainer to maintain the stability of the median palatal suture width during its interdigitation, thus preventing relapse [33,34]. The correct transverse arch width is defined as the upper teeth’s cutting cusps positioned buccally to the antagonistic teeth, and the lower teeth’s stamp cusps located at the central fossa of the antagonistic upper teeth.

Once the expander was removed, a removable retainer was used during night for additional 6 months. Specifically, patients using the Hyrax device used a Hawley plate as retention, while patients using the McNamara appliance used the same device as retention if it was intact after decementation (alternatively, a Hawley plate was fabricated). During this time interval, patients were monitored every 4–6 weeks: in case of relapse, the Hawley plate was activated.

### 2.4. CBCT Evaluation

Each patient underwent two panoramic X-rays, lateral cephalometric radiographies and CBCT scans: one at T0, performed 0 to 14 days before cementation of the orthodontic appliance; another at T1 (12 ± 2 months after T0), at the end of removable retention.

All CBCT scans were performed by a certified radiologist at the Radiology Department of the G.B. Rossi University Hospital in Verona using the NewTomVGievo device (NEWTOM, Cefla S.C., Bologna, Italy).

Parameters related to CBCT scans performed for the study were as follows: Number of axial images: 474; Axial step: 0.3 mm; Axial thickness: 0.3 mm; FSV: 110 kV; FSV: 3.00 mA; SSV: 110 kV; SSV: 3.20 mA; FOV: 16 × 16; Exposure time: 1.8 s; mAs: 5.45; Air Kerma: 2.3 mGy; DAP: 561.76 mGy × cm; DLP (head): 37.09 mGy × cm; CTD1w (head): 2.13 mGy; CTD1vol (head): 2.13 mGy.

For evaluating the morphology and dimensions of the upper airways, the natural head position (NHP) was recommended [35]. Therefore, patients were positioned upright during the scan with their chin supported by an adjustable rest and the Frankfurt plane parallel to the ground.

### 2.5. Volumetric Measurement Protocol

Volumes of the nasal cavities were collected from the CBCT scans following the method described by Kim et al. [36]; volumes of the retro-palatal and retro-glossal airways followed the method of Chang et al. [37].

**Figure 1:**
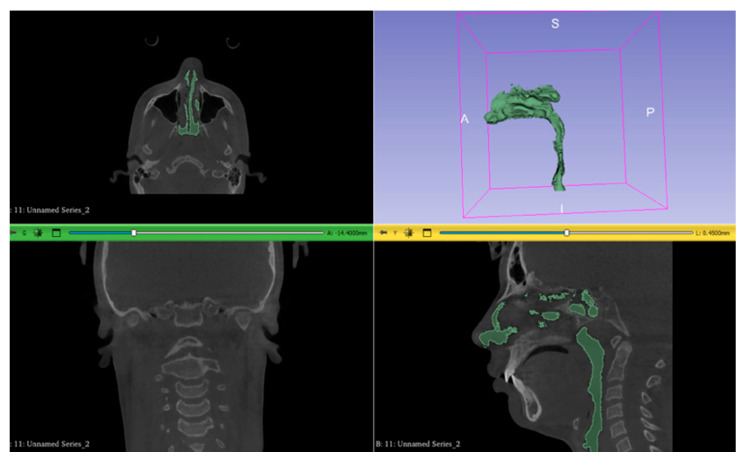
Upper airways segmentations without external air from the nostrils and maxillary sinuses and the 3D model obtained from 3D-Slicer software.

The material used for nasal cavity analysis consisted of CT scans with appropriate spatial resolution. The series of axial CT slices was imported into the 3D-Slicer software (version 5.8.1) [38]. A segmentation of the upper airways was created using radiographic intensity values for air, setting a standardized threshold value in line with other studies in literature [39] regarding accuracy of virtual rhinomanometry (Otsu-type threshold option in 3D-Slicer software with a minimum value of −1000 and a maximum value of −400 HU, Hounsfield units) [39,40]. External air from the nostrils and maxillary sinuses was removed (using the “Scissors” or “Erase” option in the 3D-Slicer software), resulting in a 3D model of the upper airways, as shown in Figure 1.

**Figure 2:**
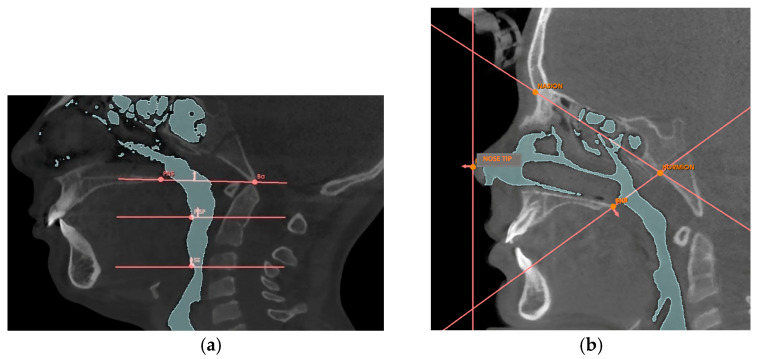
Landmarks and planes for identifying volumes. The figures illustrate: the points PNS, Nose Tip, Hormion, Nasion, Ba, PISP, SE (**a**); the corresponding planes: Hormion-Nasion, Hormion-PNS, Nasal Perpendicular, Ba-PNS, Soft palate plane, and Epiglottis plane (**b**).

After reorienting the CBCT image on the sagittal and coronal planes according to the Frankfurt plane, a series of reference points were identified on the sagittal plane, to obtain 3D models of the nasal cavity, the retro-palatine and retro-glossal airways, and to identify the reference planes (see Table A1 and Table A2 in Appendix A.2). After removing any artifacts and refining the desired area, the nasal cavity was defined as the region bounded anteriorly by the Nasal Perpendicular plane, superiorly by the Nasion-Hormion plane, and posteriorly by the Hormion-PNS plane (see Figure 2).

**Figure 3:**
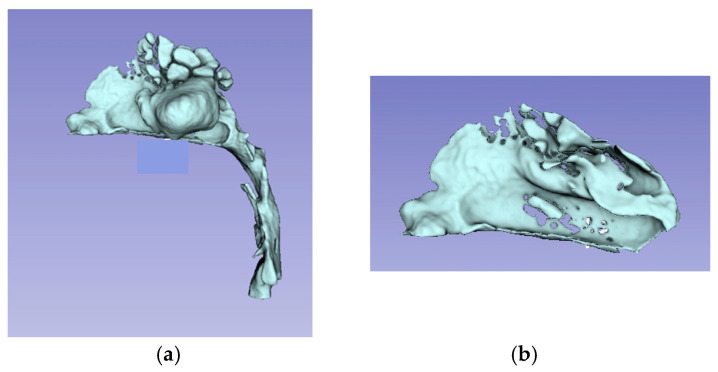
3D view of the model of upper airways (**a**); Sagittal view of the 3D model of the nasal cavity (**b**).

The unsegmented 3D model of the upper airways and the nasal cavity model derived from the intersection of the planes is shown in Figure 3.

Starting from the final 3D model, it was possible to derive the volume of the nasal cavity in cubic millimeters.

**Figure 4:**
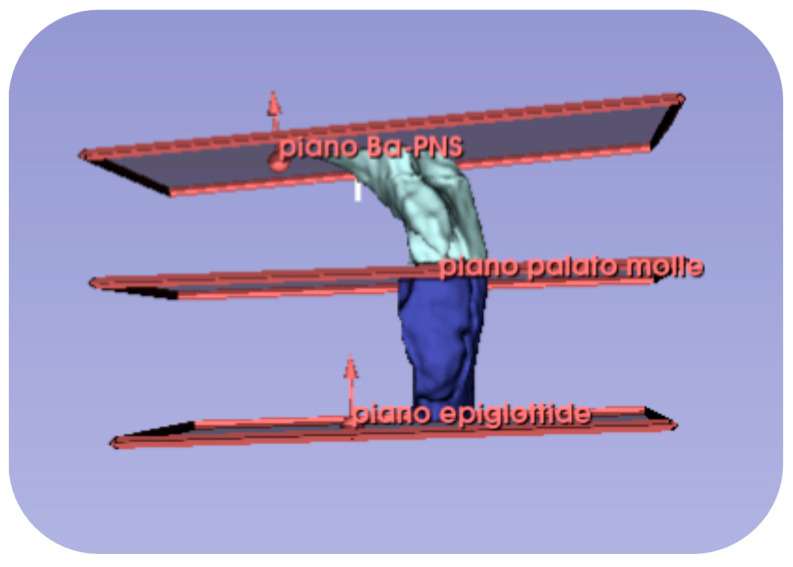
3D model of the subdivision of the retro-palatal and retro-glossal volume segmentation.

The retro-palatal cavity was defined as being bounded superiorly by the Ba-PNS plane and inferiorly by the soft palate plane, while the retro-glossal cavity was defined as being bounded superiorly by the soft palate plane and inferiorly by the epiglottis plane, as shown in Figure 2b and Figure 4.

### 2.6. Airflow Velocity Calculation Protocol

To estimate changes in airflow velocity [41,42,43,44,45], specific anatomical structures within the nasal, retro-palatal, and retro-glossal cavities were identified where airflow occurs.

A pilot simplified “hydraulic system“ was idealized by the authors to approximate, as accurate as possible, the abovementioned structures to interconnected models of two rotational solids, a cylinder (with diameter and height) and a truncated cone (with upper and lower base diameters and height), for the calculation of internal cross-sections.

**Figure 5:**
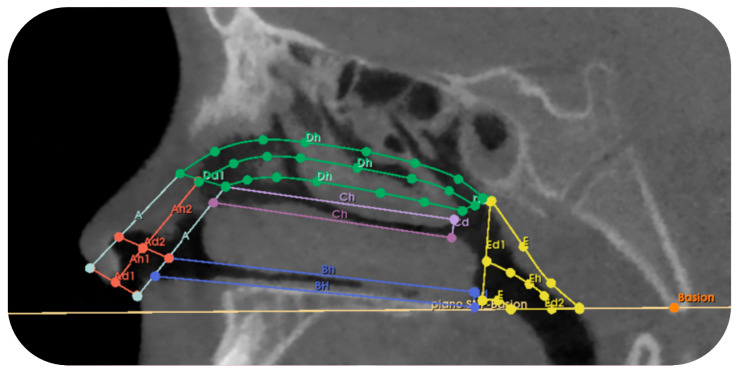
CBCT visualization of the subdivision of the nasal cavity with the respective references and measurements for the study of airflow: diameter (Ad1) and height (Ah1) of solid A1 and the height (Ah2) of solid A2; length (Bh) and diameter (Bd) of solid B; length (Ch) and diameter (Cd); length (Dh) and diameter (Dd2) of solid D; diameter 1 (Ed1), diameter 2 (Ed2), and height (Eh) of solid E.

The system identified within the nasal cavity follows the subdivision of connected references (see Table A3 in Appendix A.2) to obtain the models by visible in the sagittal CBCT projection shown in Figure 5. Specifically, the upper base of solid A1 coincides with the lower base of solid A2, and the upper base of solid A2 coincides with the lower base of solid D. Therefore, the diameters of the bases of section A1 are equal to the diameters of section A2 and the lower diameter (Dd1) of section D.

**Figure 6:**
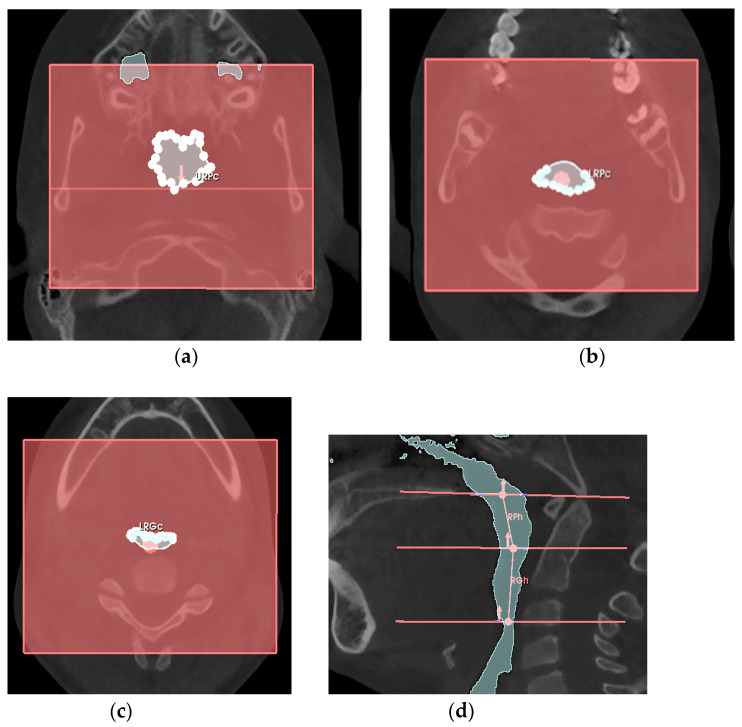
Axial view of regions of retro-palatal and retro-glossal sections: axial view of the URPc (**a**); axial view of the LRPc (**b**); axial view of the LRGc (**c**); sagittal view of the heights RPh and RGh (**d**).

Regarding the system identified within the retro-palatal and retro-glossal cavities, two trunk-cones were also determined by a series of measurements (see Table A4 in Appendix A.2) shown in Figure 6.

In this case, the lower base of the trunk-cone F coincides with the upper base of the trunk-cone G, and furthermore, the upper base of F coincides with the lower base of the previously described E section of the nasal cavity.

At this point, the airway conduit from the Ba-PNS plane to the epiglottis plane was approximated to a series of two hollow cylindrical tubes, therefore:

- the retro-palatal volume section was modeled as a right truncated cone, F, with an upper circumference URPc (from which the diameter URPd = URPc/π was derived), a lower circumference LRPc (from which the diameter LRPd = LRPc/π was derived), and a height RPh;
- the retro-glossal volume section was modeled as a right truncated cone, G, with an upper circumference LRPc (from which the diameter LRPd = LRPc/π was derived), a lower circumference LRGc (from which the diameter LRGd = LRGc/π was derived), and a height RGh.

These measurements were then entered into an Excel file with iterative calculations based on the pilot hydraulic system representing an approximation of the nasal cavity, and the retro-palatal and retro-glossal cavities, to provide an approximate mathematical estimation of the variation in airflow velocity within the hydraulic system.

Regarding the variation in airflow for each model, it is primarily based on the principle of mass conservation and the generalized Bernoulli equation. According to the principle of mass conservation, the change in mass per unit of time (flow rate, measured in m^3^/s) in a system is given by the difference between the sum of incoming flow rates and the sum of outgoing flow rates. For a closed system, this difference is zero, meaning that the total incoming mass flow rate equals the total outgoing mass flow rate.

Furthermore, the generalized Bernoulli equation describes an ideal fluid in the following form:

$$\frac{p_{i}}{\rho g}+\frac{v_{i}^{2}}{2g}+z_{i}=\frac{p_{u}}{\rho g}+\frac{v_{o}^{2}}{2g}+z_{u}+pressure\;losses$$

where: vi (inlet velocity), vo (outlet velocity), pi (inlet pressure), po (outlet pressure), ρ (constant density), zi (height loss at inlet compared to sea level), and zo (height loss at outlet compared to sea level).

Considering the data collected in this study, the value of z was set at zero, since the distance between the inlet and outlet of the tube was less than 30 m. According to this equation, any increase in velocity simultaneously results in a decrease in pressure or a change in the potential energy of the fluid.

The studied system accounts for both distributed and concentrated pressure losses: the former are due to the fact that the internal surface of the tube is not perfectly smooth, meaning the fluid encounters resistance as it flows through; the latter arise from the fact that the tube is not perfectly straight and may present changes in shape, such as abrupt expansions/contractions, sharp/wide curves, or T-junctions, all of which represent challenges for the fluid as it passes through the tube.

Standardized values for external air temperature, external air pressure, air density, kinematic viscosity, and PNIF (peak nasal inspiratory flow) were derived from the literature for each age considered [46]. The standardized and constant values across the patients in the study are: gravitational constant: 9.8 m/s; external temperature: 293.15 K (20 °C); external pressure: 101,325 Pa; fluid density: 1.2 kg/m^3^; kinematic viscosity: 1.6 × 10^−5^.

By inputting the measurements obtained from the CBCT and the standardized parameters into the iterative calculation Excel sheet, information on the variation in flow velocity at both time T0 and time T1 was obtained for both group A and group B patients, as pilot mathematical estimates rather than physical/instrumental measurements, reflecting potential changes in velocity within a section whose dimensions were modified between T0 and T1.

### 2.7. Sample Size, Data Collection and Analysis

Being a retrospective study, sample size was based on feasibility criteria, according to the medical records possible to evaluate. However, a hypothetical estimation of a sample, also for further future analysis, was performed, defining the nasal cavity volume (VN, mm^3^) as the primary response variable, measured between baseline and follow-up. In the chosen study [47], the control group is not present, so data related to VN in treated patients were considered as representative of the trend, with a difference of 200 mm^3^ hypothesized in favor of non-treated subjects. The calculation for a Student t-test for unpaired data, with α equal to 0.05 and power 0.80, leads to an estimate of the sample size of 16 patients, which can be adjusted to 18 patients including also a 10% of possible drop-out.

Data regarding the volume of the nasal, retro-glossal and retro-palatal airways were collected from the CBCT scans at T0 and T1. The airflow rate within the nasal cavities, retro-palatal airways, and retro-glossal airways was then assessed at T0 and T1. Measurements were taken by an operator not involved in the treatment of patients, using 3D-Slicer software [38] and assessing values to the nearest 0.01 mm^3^ (for volume) or m/s (for air speed). To ensure reproducibility of the reference points used for data collection, each measurement was taken twice by the same operator for all CBCTs at both T0 and T1. It was subsequently verified that there were no statistically significant differences between the two sets of data. Additionally, the operator was blinded as to whether the images were pre- or post-treatment during both measurements. Precisely, before the start of the study, the operator was calibrated for adequate intra-examiner levels of accuracy and reproducibility in recording parameters. Five cases were utilized for this purpose: for each parameter, duplicate measurements were collected with an interval of 24 h between the first and second recordings. The intra-class correlation coefficients, used as a measure of intra-examiner reproducibility, had to be greater than 0.8. Furthermore, the abovementioned exercise, according to the same method, was repeated by another another operator, different from the previous one (always not involved in the treatments of patients) for inter-examiner reproducibility.

The collected data were collected with Microsoft Excel and then analyzed using Stata v.13.0 for Macintosh (StataCorp, College Station, TX, USA). Parametric and non-parametric statistics were employed, performing: the paired Student’s *t*-test or Wilcoxon matched-pairs signed-rank test for comparing T0 and T1 within each group; the unpaired Student’s *t*-test or Wilcoxon-Mann-Whitney rank sum test to compare group A and B. Significance level was set at 0.05. Furthermore, comparisons regarding groups and time-intervals were performed considering also age ranges and patients’ growth, even if, being the study retrospective, it was not possible to match groups for these variables.

## 3. Results

Regarding the age of both patients groups at T0:

- group A consisted in 16 patients: 8 females and 8 males; 8 patients treated with the McNamara device and 8 with the Hyrax device, with an average age of 9.34 ± 1.3 years;
- group B consisted in 8 patients: 3 females and 5 males, with an average age of 11.11 ± 1.47 years.

Regarding mean age, a statistically significant difference (*p* = 0.01 *) was found between groups, suggesting that group B is closer to the pubertal growth peak.

Table 1 and Table 2 present, respectively for group A (Table 1) and group B (Table 2), mean values at T0, and mean variations between T0 and T1, for all variables measured for airway space and flow.

Analysis between T0 and T1 in group A revealed significant variations for all variables under examination. Specifically:

- a statistically significant increase can be observed for volumes VN, VRP and VRG;
- a statistically significant decrease can be observed for all variables related to airflow velocity.

Analysis between T0 and T1 in group B revealed significant variations for most variables under examination. Specifically:

- a statistically significant increase can be observed for VN;
- a statistically significant decrease can be observed for all variables related to airflow velocity;
- a not significant increase can be observed for VRP and VRG.

Finally, comparison regarding variations between T0 and T1 found a statistically significant difference between group A and B only for VN (see Table 3).

Being the study retrospective, even if it was not possible to match groups for age and patients’ growth, a further analysis, reported in Table 3, was conducted according to:

- age ranges (two age-intervals of 8–9 years and 10–12 years);
- patients’ growth, considering if stage CS varied or not between T0 and T1 (from CS1 to CS2, or from CS2 to CS3), only for group A (as in this group CS was evaluated at the first visit).

No significant differences were found, for any of the variations between T0 and T1 considered:

- between patients of 8–9 years and 10–12 years;
- for group A, between patients in which CS varied or patients in which CS did not vary.

## 4. Discussion

The aim of this observational clinical study was to evaluate, using CBCT, the volumetric changes and airflow velocity changes in the nasal cavities, retro-palatal and retro-glossal airways, resulting from the treatment with RPE with dental anchorage (group A), comparing data with a group non treated with RPE (group B).

The main resistance encountered in opening the midpalatal suture comes from the surrounding structures, such as the sphenoid bone and the zygomatic bone. Various animal studies show [12] that the complexity of interdigitation is greater in the zygomatic-maxillary suture compared to the zygomatic-temporal and zygomatic-frontal sutures. Overall, the transverse palatine suture shows the highest rate of opening compared to all circum-maxillary sutures.

Another obstacle in palatal expansion is the rigid interdigitation that can occur even in mixed-late dentition of the pterygomaxillary and pterygopalatine sutures [2], leading to fan-shaped opening of the suture in the horizontal plane. For this reason, treatment is preferable in patients still growing [14], before the ossification of the sutures (ideally before puberty, under 15 years of age). To achieve better stability, hypercorrection of maxillary expansion is planned, as a recurrence of at least one-third is expected. Additionally, to minimize recurrence, the use of removable or fixed orthodontic appliances for at least 3 months is indicated. Skeletal effects of expansion remain only if a retention period of at least 12 months is performed [12] (6 months of fixed retention followed by 6 months of removable night retention), during which ossification of the space obtained during suture opening occurs.

The effects of RPE on breathing depend on the cause, location, and severity of nasal obstruction, so there can be null, minimal, or marked changes [10]. Some authors [48,49] argue that a reduction in nasal resistance is not significantly observed in all patients treated with RPE, but mainly in a subgroup of patients with initial resistance values > 5.5 cm H_2_O/L/s, with an overall effect of increase in intra-nasal capacity of 8–10 mm at the level of the inferior turbinates. On the other hand, it was also reported [18] a reduction in nasal airway resistance of 45–53% with RPE, maintained after removal of the orthodontic device. Moreover, the maximum negative pressure values regarding the pharyngeal airway decrease after RPE treatment, leading to a reduction in the constriction of the oropharyngeal airway, and thus improving pharyngeal ventilation [50].

In the light of this heterogeneous evidence, it is still debated whether RPE can be considered a therapeutic device for respiratory disorders [51]. According to other authors [52], the volume of the nasal cavity can be significantly affected by the treatment, as maxillary bones, forming half of the anatomical structure of the nasal cavity, influence both the anatomy and physiology of the nose through the separation of the mid-palatal suture. In the present study, a statistically significant increase was found in the variation of VN between T0 and T1, both for patients treated with RPE (median value 1971.01 mm^3^, *p* < 0.001) and patients not treated (median value of 1005.39, *p* = 0.01).

Regarding variation of nasal cavity volume, the present outcomes are in line with other similar investigations in literature, which reported values of 1419.47 mm^3^ (15 subjects with an average age of 13.86 years) [52], and 1646.1 mm^3^ (25 patients with an average age of 10.5 years, analyzed by CBCT, with a diagnosis of oral respiration) also associated with improvements in breathing and quality of life [53]. Other greater measurements of 1868 mm^3^ (20 patients with an average age of 12.3 years) [47] and 1886 mm^3^ (18 patients with an average age of 14.7 years undergoing orthodontic-orthopedic treatment) [54] were reported.

Moreover, a systematic review of 27 studies [55] did not specify the limits of the nasal cavity considered, evidencing on CBCT an average variation of 1604 mm^3^ in nasal cavity volume between 3 and 12 months after expansion. Another systematic review of 17 studies [56] observed an increase of 1218.3 mm^3^ in nasal cavity volume between baseline and 8 months after treatment, evidencing that the main limitation of studies is the lack of control cases, which did not account for normal growth effects. Similarly, Si et al. [57] demonstrated a nasal cavity volumetric variation of 2236.55 mm^3^ on CBCT from T0 to T1 in 35 children aged 6 to 10 years, but the lack of a control group for comparison was again evidenced in determining whether the volume changes were due to growth, the expander, or both.

Regarding the retro-palatal and retro-glossal areas, our study found a statistically significant increase between T0 and T1 in both volumes (median value of 340.26 mm^3^, *p* = 0.001; median value of 503.77 mm^3^, *p* = 0.002) in group A. Outcomes reported for VRP by other authors differently vary, with values of 1413.9 mm^3^ [58], 1273.1 mm^3^ [59], 1201.2 mm^3^ [37], 871 mm^3^ [60], 331.41 mm^3^ [61], 239.36 mm^3^ [62] and 100 mm^3^ [63]. The same for VRG, with values of 1601.4 mm^3^ [58], 988 mm^3^ [60], 533.8 mm^3^ [37], 239.36 mm^3^ [62] and 93.34 mm^3^ [61]. Increase of VRP and VRG is justifiable by the opening of the mid-palatal suture due to the rapid palatal expander, leading to an increase in nasal cavity volume and a decrease in nasal resistance, resulting in improved physiological nasal breathing. Along with this improvement, there is a decrease in the maximum negative pressure of the pharyngeal airways, making them less prone to constriction. This, combined with the physiological growth of the individual, leads to an appreciable increase in retro-palatal and retro-glossal airways after treatment with RPE [50,62,64]. However, consistently with other investigations [58,59,60,61,62,63,65], our comparison between groups did not find significant differences in these volumes. From these outcomes, it can be deduced that the increase in retro-palatal and retro-glossal volumes can be likely more influenced by patients’ growth rather than treatment with the RPE. Nevertheless, the retrospective study design did not allow to match groups for patients’ growth, and a further analysis for group A did not find any significant differences between patients in which CS varied or patients in which CS did not vary. Same as for analysis of age ranges (8–9 and 10–12 years). Authors thus hypothesize the influence of patients’ growth as clinically relevant for the outcomes of the study, as natural growth and anatomical remodeling are very important variables in determining the final anatomy of an individual’s airways and consequently their respiratory capacity [66]. The presence of a control group in which was not possible to retrospectively evaluate CS stage does not otherwise allow for definitive conclusion whether the volume changes were due to growth, the expander, or both [57]. However, it is important to note that in the control group, the growth variable seems to have a more significant impact, as the mean age of the control group is close to the pubertal growth peak.

The estimation of airflow velocity in standardized conditions carried out in our study provides an indication of how the airflow velocity within the nose changes based on variations in the anatomical structures of the nasal cavity itself, with the aim of analyzing any potential increase in functionality. The results obtained for each nasal section considered show a statistically significant reduction in velocity between T0 and T1 for both groups, while the difference between the two groups was not statistically significant.

While the association between anatomical changes in the nasal cavity and treatment with the RPE is well-studied in the literature, opinions on the actual change in airflow in patients treated with RPE on teeth are highly controversial. An attempt to use theoretical principles of hydraulics was based on nasal cavity measurements to study airflow variations [67], where airflow was calculated as a variation of pressures and volumes, whereas our study describes it as a variation in velocity. Even today, acoustic rhinometry is more commonly used as a tool for investigating nasal functionality, but some authors [41] report that nasal patency does not necessarily correspond to subjective symptoms of nasal obstruction. Recently [68], experimental and computer-based methods based on CBCT to evaluate nasal airflow have been studied, although the anatomical complexity considered may create a barrier to obtaining accurate results.

Several studies by various authors have analyzed the variations in airflow within the nasal cavity following treatment with a rapid palatal expander, along with studies identifying various challenges in evaluating nasal airflow. Iwasaki et al. [50] studied 22 patients (age range 10–16 years) to assess the changes in nasal ventilation using a computerized fluid dynamics software. They found a reduction in airflow velocity (measured in m/s) from an average of 25 m/s to 17 m/s post-treatment (a 30% decrease). The authors noted that not all patients respond similarly to treatment with a tooth-borne rapid palatal expander (RPE), for patient-related factors (such as the complex structure of nasal airways, hypertrophy of nasal mucosa, adenoids, etc.) and treatment-related factors (expander design, amount of expansion, age, skeletal maturity, activation protocol, retention period, evaluation method, etc.), with a possible contribution of airway growth.

Wertz et al. [43] found no statistically significant difference compared to T0 in a group of 9 patients who did not present breathing abnormalities before treatment with an expander. They hypothesized that expansion likely occurs mainly in the anterior and lower regions of the nasal cavity: consequently, changes in nasal airflow are not apparent if there is an obstruction in the posterior region, which is less affected by the orthodontic-orthopedic device.

Chen et al. observed [44], assessing CBCT on 15 patients (average age 9.57 years), that, after treatment with a RPE, airflow tends to become more symmetrical between the right and left sides and faster along the lower part of the turbinates, which is the region mainly involved in the mucosal exchange. As velocity decreases allows more time for contact with the nasal mucosa and facilitate air warming/cooling and humidification, authors suggested that the reduction in velocity lessens the stress on the nasal cavity walls, leading to less irritation of the blood vessels and enhancing the protective role of the nasal mucosal membrane.

In the treated group, between T0 and T1, there was a statistically significant decrease in retro-palatal airflow (median value of −0.75 m/s) and a statistically significant decrease in retro-glossal airflow (median value of −0.99 m/s). In the control group, between T0 and T, there was also a statistically significant reduction in retro-palatal airflow (median value of −0.74 m/s) and a statistically significant decrease in retro-glossal airflow (median value of −1.29 m/s). Only a few studies in address these variables, as interest in the dimensional and functional changes of the pharyngeal airways is recent, and the methodologies used to collect data differ. Iwasaki et al. [58] used volumetric rendering software (INTAGE Volume Editor; CYBERNET, Tokyo, Japan) to create 3D models of the air cavities and the hard and soft tissues of the craniofacial region based on CBCT images. They then simulated upper airway ventilation using fluid dynamics software (PHOENICS; CHAM-Japan, Tokyo, Japan). In the group treated with a rapid palatal expander, they found a statistically significant reduction in maximum airflow velocity compared to T0 by −2.68 ± 5.19 m/s. Iwasaki et al. [50] in another study, using the same software mentioned in the previous study, simulated airflow pressure within the nasal and pharyngeal cavities, analyzing nasal resistance and maximum negative pressure in the pharyngeal airways in patients treated with a rapid palatal expander. This study found significantly lower maximum negative pressure values in the pharyngeal region post-treatment with RPE. Fastuca et al. [45] used the freeware software Project Falcon 2013 by Autodesk^®^, a wind tunnel simulator, to investigate interactively how upper airway aerodynamics, such as airflow quality and velocity, change after treatment with RPE, considering a single volume extending from the nostrils to the lower limit of the pharynx as seen in CBCT images. In their simulation, air flowing through the choanae toward the nasopharynx encounters changes depending on the resistance due to volume and the path followed. Pre-treatment, the airflow gradually becomes more turbulent as it enters, increasing in velocity in certain areas, especially in the lower part, while remaining stationary in others due to a restricted volume and smaller diameter that partially blocks the incoming airflow. After RPE treatment, the airflow becomes more stable and regular in velocity, as the enlargement of the pharyngeal volume prevents the airflow from being blocked when it hits the posterior airway wall, resulting in no changes in velocity or path.

The results obtained in the treated group in this study are thus consistent with the literature: the reduction in airflow velocity compared to T0 is presumably due to the fact that following the opening of the median palatal suture, there is an increase in the volume of the nasal and pharyngeal cavities, a decrease in nasal cavity resistance, and a decrease in maximum pharyngeal negative pressure, making the pharyngeal airways less prone to constriction compared to T0 [45,50]. These changes favor the transition from oral to physiological nasal breathing, with consequent benefits for proper craniofacial development and nasal-respiratory disorders (allergies, recurrent rhinitis, otitis).

The effects of tooth-borne RPE on the airways have often been described through the reduction of nasal resistance, achieved through maxillary repositioning, which leads to the opening of the nasal valves located between the superior and inferior cartilages and the piriform apertures [1,57,69,70,71,72,73,74]. In the study by De Felippe et al. [73], using acoustic rhinometry, the authors identified a significant reduction in nasal airway resistance in patients treated with various types of maxillary expansion devices. However, in their long-term follow-up study [75], it was found that the mean values of these parameters were in line with those of the controls (subjects who had never undergone orthodontic treatment). It was concluded that tooth-borne RPE may help normalize parameters in treated patients but does not cause long-term benefits. Natural growth and remodeling, as mentioned by the authors, are likely very important variables in determining the final anatomy of an individual’s airways and thus modifying respiratory capacity. However, the results of these studies show that anatomical changes provided by tooth-borne RPE do not seem to have any effect on the final product of growth, at least regarding function. Hartgerink et al. [72] found a reduction in nasal resistance in both the treated and control groups using the “SNORT” (Simultaneous Nasal and Oral Respirometric Technique) and suggested that growth and development might also contribute to reducing nasal resistance. The authors indicated that the forces resulting from the expansion probably induce remodeling of the nasal cavity bones, combined with the effect of growth, where there is a phenomenon of lymphatic tissue atrophy. Furthermore, a significant component of inter-individual variability was highlighted. Torre et al. found a significant increase in PNIF in 44 children (mean age 10.57 ± 1.93 years) treated with a rapid palatal expander compared to the control group, noting an improvement in oral breathing. The authors also identified difficulty in analyzing the different studies describing the effect of tooth-borne RPE and variations in airflow due to the different methods used in each study, which are difficult to compare and not yet standardized today [76].

The recent interest in studying changes in pharyngeal volumes after treatment with rapid palatal expansion is due to the hypothesis of potential benefits that this treatment may provide in patients with respiratory disorders. Various studies show that after RPE treatment in these patients, a physiological nasal airflow is restored [77,78,79,80], which generates a lower inspiratory sub-atmospheric pressure that reduces vulnerability to pharyngeal collapse [77], thereby also improving pharyngeal ventilation. The improvements induced by RPE in patients with respiratory disorders are therefore more functional in nature, as there is still no strong evidence supporting the hypothesis that treatment with this device leads to an increase in the volume of the oropharyngeal airways [63]. Although increases in the size and function of the upper airways have been observed after RPE, in terms of improved nasal breathing, the use of the expander is not indicated solely for this purpose [63,81,82] and its application remains specific to the correction of transverse palatal deficiencies.

Future research could test the impact of rapid palatal expansion on specific conditions such as obstructive sleep apnea (with or without ENT intervention). In general, both subjective patient evaluations and the various methods for measuring nasal airflow and nasal airway resistance (NAR) show that there is a significant improvement in nasal breathing after maxillary expansion, but the large variation in individual responses to tooth-borne RPE and the results from the study and current literature suggest caution.

### Study Limitations

The retrospective observational nature of the present study affects sample and age distribution, with groups not possible to match for age and growth.

Moreover, lack of standardization regarding the assessment of pre-treatment breathing, respiratory phase, tongue posture and swallowing are present. Given the great anatomical and functional complexity of the airways, long-term changes resulting from RPE on airway dimensions and functions have been studied using various methods (MRI, acoustic rhinometry, X-rays, and CBCT), which may differ in determining the actual changes in the nasal cavity and respiratory function [83].

Regarding methodology, the use of different software for calculating airway volume and flow, a lack of standardization of the planes delimiting the nasal cavity and pharyngeal volumes, and the assumption of a constant flow in rigid-walled cavities with constant air pressure and temperature, all reveal an approximation for the pilot model for experimental setting, which needs to be validated.

Additionally, although the pilot model used in this study for flow evaluation (consisting of an iterative Excel file developed based on hydraulic laws and anatomical variations) represents a quick and cost-effective method, it is not easily usable and not properly reproducible, with a high degree of approximation that can affect the reliability of the obtained results. Future developments for more accurate and precise flow calculations therefore include the use of computational fluid dynamics (CFD), recognized as a new high-tech tool useful for improving and expanding knowledge regarding airflow in the upper airways after orthodontic-orthopedic treatment and the inclusion of other parameters characterizing respiratory function [23,45,82].

## 5. Conclusions

Within the limitations of this retrospective pilot study, the treatment with RPE for maxillary deficiency revealed promising outcomes, with significant increase in retro-palatal, retro-glossal and nasal volumes, and significant changes in airflow velocities. The evaluation allowed for comparison with a control group, which was not significantly different, except for a lower increase in VN. To assess the stability of results and strengthen evidence on the effects of RPE on airway functions in orthodontics and dentofacial orthopedics, further randomized controlled trials and long-term double-blind studies (with evaluation after the pubertal growth peak), with larger sample size, are needed.

## Figures and Tables

**Table 1:** **Group A:** mean measurements at T0 and at T1 and their variations between T0 and T1, expressed as mean ± SD (standard deviation), or *median (minimum, maximum)*, for all variables assessed for airway space and flow.

Variable	Group A (Treated with RPE)				
	Average Values at T0		Average Variations Between T0 and T1		Comparison Between T0 and T1
	Mean	SD	Mean/*Median*	SD/*(min; max)*	*p* Value
VN (mm^3^)	10,097.22	2225.9	*1971.01*	*1275.8; 3431.64*	<0.001 *
VRP (mm^3^)	3533.17	516.37	*340.26*	*−173.13; 1664.13*	0.001 *
VRG (mm^3^)	2679.81	470.21	*503.77*	*−124; 1892.81*	0.002 *
S_A1_R (m/s)	10.68	2.53	−2.89	1.46	<0.001 *
S_A1_L (m/s)	10.79	2.58	−3.31	2.21	<0.001 *
S_A2_R (m/s)	8.26	1.74	−2.32	1.19	<0.001 *
S_A2_L (m/s)	7.92	1.54	−2.18	1.2	<0.001 *
S_B_R (m/s)	7.9	1.36	−2.54	0.67	<0.001 *
S_B_L (m/s)	7.98	1.31	−2.56	0.77	<0.001 *
S_C_R (m/s)	7.01	1.17	−2.31	0.69	<0.001 *
S_C_L (m/s)	6.9	1.19	−2.16	0.76	<0.001 *
S_D_R (m/s)	11.45	2.05	−3.93	0.98	<0.001 *
S_D_L (m/s)	11.33	2.14	−3.72	1.02	<0.001 *
S_E (m/s)	4.3	0.71	−0.8	0.61	<0.001 *
S_RP (m/s)	4.53	1.01	*−0.75*	*−2.29; 0.06*	<0.001 *
S_RG (m/s)	6.24	1.99	*−0.99*	*−4.33; −0.09*	<0.001 *

* = statistically significant difference in time-intervals; VN = nasal cavity volume; VRP = retropalatal volume; VGR = retroglossal volume; S_A/B/C/D/E = speed values of simulated flow in the subdivisions of the nasal cavity into sections A-B-C-D-E (right = R and left = L); S_RP = speed values of retropalatal flow; S_RG = speed values of retroglossal flow.

**Table 2:** **Group B:** mean measurements at T0 and at T1 and their variations between T0 and T1, expressed as mean ± SD (standard deviation), or *median (minimum, maximum)*, for all variables assessed for airway space and flow.

Variable	Group B (Control)				
	Average Values at T0		Average Variations Between T0 and T1		Comparison Between T0 and T1
	Mean	SD	Mean/*Median*	SD/*(min; max)*	*p* Value
VN (mm^3^)	9854.72	1445.70	*1005.39*	*560.7; 1283.95*	0.01 *
VRP (mm^3^)	3973.25	876.63	*64.135*	*−112.57; 784.44*	0.26
VRG (mm^3^)	2977.32	751.83	*83.13*	*−100.95; 920.10*	0.12
S_A1_R (m/s)	12.93	1.96	−3.39	1.81	0.01 *
S_A1_L (m/s)	13.2	1.96	−3.65	2.1	0.001 *
S_A2_R (m/s)	9.81	1.36	−2.58	1.29	<0.001 *
S_A2_L (m/s)	9.59	1.64	−2.38	1.27	<0.001 *
S_B_R (m/s)	7.84	1.38	−2.23	0.79	<0.001 *
S_B_L (m/s)	8.9	0.95	−2.14	0.76	<0.001 *
S_C_R (m/s)	8.00	0.96	−2.00	0.67	<0.001 *
S_C_L (m/s)	6.89	1.22	−2.16	0.76	<0.001 *
S_D_R (m/s)	12.83	1.74	−3.93	0.98	<0.001 *
S_D_L (m/s)	12.32	1.54	−3.72	1.02	<0.001 *
S_E (m/s)	4.01	0.58	−0.8	0.61	<0.001 *
S_RP (m/s)	3.58	0.54	*−0.74*	*−1.05; −0.16*	0.01 *
S_RG (m/s)	4.73	0.87	*−1.29*	*−1.87; 0.38*	0.02 *

* = statistically significant difference in time-intervals; VN = nasal cavity volume; VRP = retropalatal volume; VGR = retroglossal volume; S_A/B/C/D/E = speed values of simulated flow in the subdivisions of the nasal cavity into sections A-B-C-D-E (right = R and left = L); S_RP = speed values of retropalatal flow; S_RG = speed values of retroglossal flow.

**Table 3:** Inter-group analysis: comparisons of variations between T0 and T1 performed according to groups of treatment, age range and CS stage.

	Inter-Group Analysis (*p* Value)		
Variable (Average Variations T0–T1)	Comparison Between Group A and Group B	Comparison Between 8–9 Years and 10–12 Years	Comparison (Only for Group A) Between Varied or Not Varied CS Stage
VN (mm^3^)	0.001 *	0.62	0.61
VRP (mm^3^)	0.08	0.88	0.95
VRG (mm^3^)	0.14	0.5	0.39
S_A1_R (m/s)	0.47	0.54	0.33
S_A1_L (m/s)	0.72	0.36	0.86
S_A2_R (m/s)	0.62	0.62	0.39
S_A2_L (m/s)	0.71	0.83	0.33
S_B_R (m/s)	0.32	0.7	0.12
S_B_L (m/s)	0.21	0.46	0.23
S_C_R (m/s)	0.30	0.36	0.28
S_C_L (m/s)	0.36	0.54	0.19
S_D_R (m/s)	0.06	0.36	0.95
S_D_L (m/s)	0.09	0.25	0.46
S_E (m/s)	0.4	0.66	0.4
S_RP (m/s)	0.75	0.89	0.56
S_RG (m/s)	0.66	0.34	0.19

* = statistically significant difference between groups of comparison; VN = nasal cavity volume; VRP = retropalatal volume; VGR = retroglossal volume; S_A/B/C/D/E = speed values of simulated flow in the subdivisions of the nasal cavity into sections A-B-C-D-E (right = R and left = L); S_RP = speed values of retropalatal flow; S_RG = speed values of retroglossal flow.

**Figure A1:**
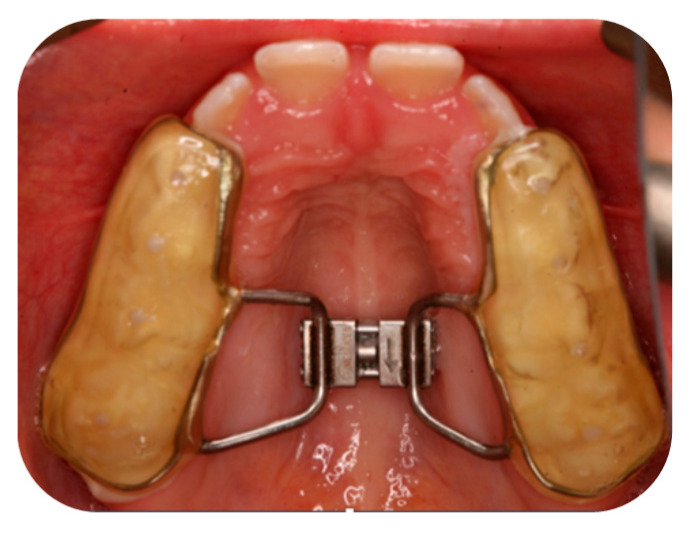
McNamara RPE device with resin splints.

**Figure A2:**
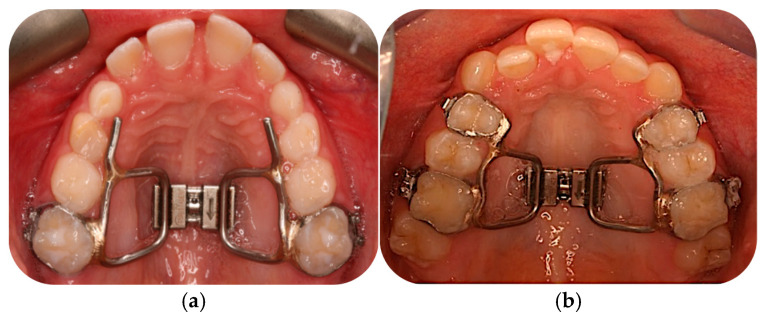
Hyrax RPE device with one cemented band on the first molars (**a**) or two bands cemented on the first molars and premolars (**b**).

## Data Availability

Data are available from the corresponding authors upon reasonable request.

## Appendix A

### Appendix A.1. Orthodontic Treatment with RPE for Group A

RPE is an orthodontic-orthopedic treatment reserved for patients diagnosed with maxillary endognathia and aims to open the midpalatal suture and circum-maxillary sutures. Expansion occurs when the forces applied to the teeth and the upper alveolar processes exceed the limits necessary for orthodontic dental movement. The applied force acts as an orthopedic force that opens the midpalatal suture by compressing the periodontal ligament, tilting the alveolar processes, and the teeth supporting the appliance, gradually opening the palatine suture. The opening of the midpalatal suture occurs non-parallel, with greater opening identifiable at the anterior nasal spine and less opening in the posterior palatal region [10,11,14]. The maxilla rotates both in the sagittal and frontal planes, mainly with a downward and forward movement [10,11]: looking at the frontal plane, the rotation fulcrum is located approximately at the fronto-maxillary suture for each maxilla, resulting in the lowering of the palatine processes of the maxilla and the palatal vault, with the descent of the bispinal plane; in the sagittal plane, the maxillary point A advances due to an increase in the bony base [11]. From a dental point of view, a diastema between the upper central incisors can be observed: the mesial inclination of the crowns occurs due to the elastic return of the transseptal fibers; once the crowns meet, the continuous pulling of the fibers causes the root movement to converge towards the original axis of inclination (about 4 months) [10].

Regarding the McNamara device [30], it is a fixed rapid palatal expander with resin splints, consisting of a central expansion screw with lateral arms welded to a steel framework extending circumferentially around the posterior upper teeth (typically from the deciduous molars to the first and/or second molars). The framework is covered by acrylic splints that cover the vestibular, occlusal, and palatal surfaces of the teeth, including the mesial surface of the deciduous molars and the distal surface of the first or second permanent molars (as shown in Figure A1).

Regarding the Hyrax device [16], it features a rectangular metal structure with a central screw, connected to orthodontic bands cemented into the first permanent molars and/or maxillary premolars (alternatively, the E element and/or first premolars) through metal arms. These arms are in contact with the palatal surfaces of the teeth, one for each hemi-arch, extending from the banded teeth to the canines and/or second molars (as shown in Figure A2).

For the group of patients treated with the McNamara expander, new alginate impressions were taken for the fabrication of the device. For the patients in the group treated with the Hyrax expander, band fitting was performed followed by new alginate impressions with the bands for the fabrication of the device. The devices were finally delivered and cemented with dual auto-photo-polymerizing glass ionomer cement (GC Fuji PLUS^®^ Radiopaque Reinforced Glass Ionomer Luting Cement). Each side was polymerized for 120 s after removing excess cement that had overflowed from the resin showers or bands.

The expansion protocol used involved four activations at the time of cementation, followed by three daily activations (two in the morning and one in the evening) until the prescription was completed. At each activation of the appliance, 1/4 turn of the screw was performed, equating to 0.2 mm (1 mm of expansion is achieved with 5 activations). The patient and parent were provided with instructions on home oral hygiene, proper nutrition, and appliance maintenance, as well as the key for performing the activations. Additionally, parents were informed about the expected formation of an inter-incisive diastema after a few days, indicating successful palatal expansion. This diastema resolves spontaneously [33] through mesial migration of the dental elements promoted by the traction of the supra-crestal gingival fibers.

### Appendix A.2. Volumetric Measurement Protocol

**Table A1:** Reference points for volume planes: definitions of the points were considered for constructing the reference planes.

Reference Point	Description
Nose tip	The most anterior point of the nose
PNS	Posterior nasal spine, the most posterior point of the hard palate
Hormion [42]	Point where the posterior border of the vomer articulates with the sphenoid bone
Nasion	The most anterior point of the fronto-nasal suture
BA	Basion, the lowest point of the anterior border of the foramen magnum
PISP	Posteroinferior point of the soft palate, the most posterior and inferior point of the soft palate
SE	Superior point of the epiglottis, the uppermost point of the epiglottis

**Table A2:** Reference planes for volume: definitions of the planes were considered to define the region of interest of the upper airways.

Planes	Description
Nasal Perpendicular	Vertical plane passing through the tip of the nose
Hormion-PNS	Plane passing through the Hormion point and the PNS point
Hormion-Nasion	Plane passing through the Hormion point and the Nasion point
Ba-SPN [43]	Plane passing through the Ba point and the PNS point
Soft palate plane [43]	Horizontal plane passing through the PISP point
Epiglottis plane [43]	Horizontal plane passing through the SE point

**Table A3:** Subdivision of Nasal Cavity Sections: description of the values recorded for each rotational solid identified within the “hydraulic system” of the nasal cavities and definition of each section of the nose taken in consideration.

Section of the Nasal Cavity	Dimensions	Section Definition
TRUNK-CONE A1 Left and right	Diameter 1 upper base and Diameter 1 lower base	It includes the vertical section of the nose that extends vertically from the entrance of the right/left nostril and ends at the upper margin of the inferior nasal concha.
	Height 1	
TRUNK CONE A2 Left and Right	Diameter 2 upper base and Diameter 2 lower base	It includes the vertical section of the nose that extends from the end of section A1 to the upper margin of the middle nasal concha.
	Height 2	
CYLINDER B Left and right	Diameter Bd	It includes the section of the nose that extends horizontally from the end of section A1 to the beginning of the choana, corresponding to the inferior nasal concha.
	Height Bh	
CYLINDER C Left and right	Diameter Cd	It includes the section that extends horizontally from the end of section A2 to the beginning of the choana, corresponding to the middle nasal concha.
	Height Ch	
TRUNK CONE D Left and Right	Diameter Dd1	It includes the section that extends horizontally from the upper margin of section A2 to the beginning of the choana, corresponding to the superior nasal concha.
	Diameter Dd2	
	Height Dh	
TRUNK CONE E	Diameter Ed1	It includes the section that extends from the junction point of sections B-C-D to the reference plane Basion-PNS.
	Diameter Ed2	
	Height Eh	

**Table A4:** Definition of the solids of rotation that constitute the nasal sections considered.

Variable	Definition
URPc (mm)	Upper circumference of the retro-palatal volume, measured on the axial slice where the Ba-PNS plane superiorly defines the retro-palatal volume.
LRPc (mm)	Lower circumference of the retro-palatal volume, measured on the axial slice where the horizontal plane of the soft palate inferiorly defines the retro-palatal volume and superiorly defines the retro-glossal volume.
LRGc (mm)	Lower circumference of the retro-glossal volume, measured on the axial slice where the horizontal plane of the epiglottis inferiorly defines the retro-glossal volume.
RPh (mm)	Height of the retro-palatal volume, the distance measured on the median sagittal slice of the retro-palatal volume from the midpoint of the upper limit defined by the Ba-PNS plane to the midpoint of the lower limit defined by the horizontal plane of the soft palate.
RGh (mm)	Height of the retro-glossal volume, the distance measured on the median sagittal slice of the retro-glossal volume from the midpoint of the upper limit defined by the horizontal plane of the soft palate to the midpoint of the lower limit defined by the horizontal plane of the epiglottis.
